# Esthetic rehabilitation of traumatic enamel defects using resin infiltration and microabrasion: a two-case report

**DOI:** 10.3389/fdmed.2026.1924961

**Published:** 2026-09-11

**Authors:** Samreena Kalander, Aravind R. Kudva, M. S. Prathap, Nihala Mariyam, Aysath Aphiya A

**Affiliations:** Department of Conservative Dentistry and Endodontics, Yenepoya (Deemed to be University) Dental College, Mangaluru, Karnataka, India

**Keywords:** case report, composite resin, dental trauma, enamel hypoplasia, microabrasion, resin infiltration

## Abstract

Localized trauma-associated developmental enamel defects and non-cavitated enamel opacities in the anterior dentition can produce marked esthetic concerns, particularly when associated with childhood trauma to primary teeth and subsequent defects in permanent successors. Conventional restorative treatment may require unnecessary removal of sound enamel, whereas minimally invasive approaches aim to preserve tooth structure while improving optical integration. This case report describes two de-identified male patients aged 17 and 18 years who presented with whitish-brown discoloration and uncomplicated crown fractures involving maxillary anterior teeth. Both patients reported a history of childhood trauma, and clinical examination revealed localized enamel hypoplasia affecting teeth 21 and 22, associated with Ellis Class I and II fractures. A staged esthetic treatment protocol was performed using rubber dam isolation, enamel microabrasion with 6.6% hydrochloric acid slurry containing silicon carbide microparticles, resin infiltration and selective direct composite restoration where required. Etch-dry cycles were repeated according to the clinical optical response before resin infiltration. Five cycles were performed in Case 1, whereas in Case 2 the sequence was repeated according to the residual opacity, although the exact number of repetitions was not documented. In both cases, the combined approach produced immediate reduction in opacity, improved enamel masking, restoration of natural morphology, and satisfactory esthetic integration with adjacent enamel. Follow-up showed stable esthetic outcomes without postoperative sensitivity, pain, or periodontal complications, and both patients reported satisfaction with the esthetic outcome of treatment. These cases demonstrate that enamel microabrasion combined with resin infiltration and selective composite augmentation can be an effective minimally invasive option for managing traumatic localized enamel hypoplasia in the esthetic zone.

## 1 Introduction

The philosophy of operative dentistry has shifted from predominantly restorative intervention toward prevention-oriented and minimally invasive care (1, 2). This approach prioritizes preservation of sound tooth structure while controlling lesion progression and improving esthetics. It is especially relevant when anterior enamel defects are non-cavitated or only superficially affected.

White spot lesions are characterized by subsurface enamel porosity beneath a relatively intact surface layer, resulting in altered light scattering and a clinically visible chalky-white opacity (3–5). Developmental enamel defects may produce a similar esthetic appearance but differ in their etiology and morphology. Trauma to primary incisors can disturb development of the permanent successors and may result in localized enamel defects that persist after eruption.

Developmental defects of enamel comprise alterations in enamel quality and/or quantity and may clinically present as demarcated opacities, diffuse opacities, hypoplasia, or combinations of these defects. Hypoplasia is a quantitative defect characterized by reduced enamel thickness and may present as pits, grooves, or partial enamel loss, whereas enamel opacity primarily represents a qualitative alteration. Post-eruptive enamel breakdown or fracture may further modify the clinical presentation. Accurate characterization of the defect is therefore important because treatment selection depends on lesion morphology, depth, surface integrity, and the extent of structural enamel loss (6).

Management should be individualized according to the clinical characteristics of the defect. Superficial discoloration may be improved by microabrasion, whereas suitable subsurface opacities with an intact enamel surface may benefit from resin infiltration. When structural loss, fracture, or surface depression is present, limited direct composite restoration may be required to restore morphology. A staged approach allows treatment to progress from surface modification and optical masking to selective restorative correction according to the clinical response.

Topical fluorides and casein phosphopeptide-amorphous calcium phosphate may promote remineralization or lesion arrest, although their ability to provide predictable esthetic masking is limited (7). Resin infiltration provides a microinvasive approach by occluding enamel microporosities with a low-viscosity resin, thereby reducing light scattering and improving optical integration with the surrounding enamel (8–10).

The present report describes the staged use of microabrasion, etch-dry optical reassessment during resin infiltration, and selective composite augmentation in two adolescents with localized trauma-associated developmental enamel defects and uncomplicated incisal fractures. Treatment was modified according to the clinical response of the lesions, with the objective of improving esthetics while limiting unnecessary restorative intervention.

## 2 Case description

This report describes two de-identified cases of localized trauma-associated developmental enamel defects involving the maxillary anterior teeth and associated with uncomplicated incisal fractures. Written informed consent for treatment and publication of clinical information and photographs was obtained in accordance with institutional requirements.

### 2.1 Case 1

A 17-year-old male patient presented with a chief complaint of brownish-white discoloration and fracture of the maxillary anterior teeth. The appearance had been present since eruption of the permanent incisors. He reported a history of falls during childhood, although the exact age at injury could not be recalled. He reported no pain, swelling, or sensitivity. The medical history was non-contributory, with no current medication use or known allergies. There was no relevant family history of developmental enamel defects or previous dental treatment involving the affected teeth.

**Figure 1:**
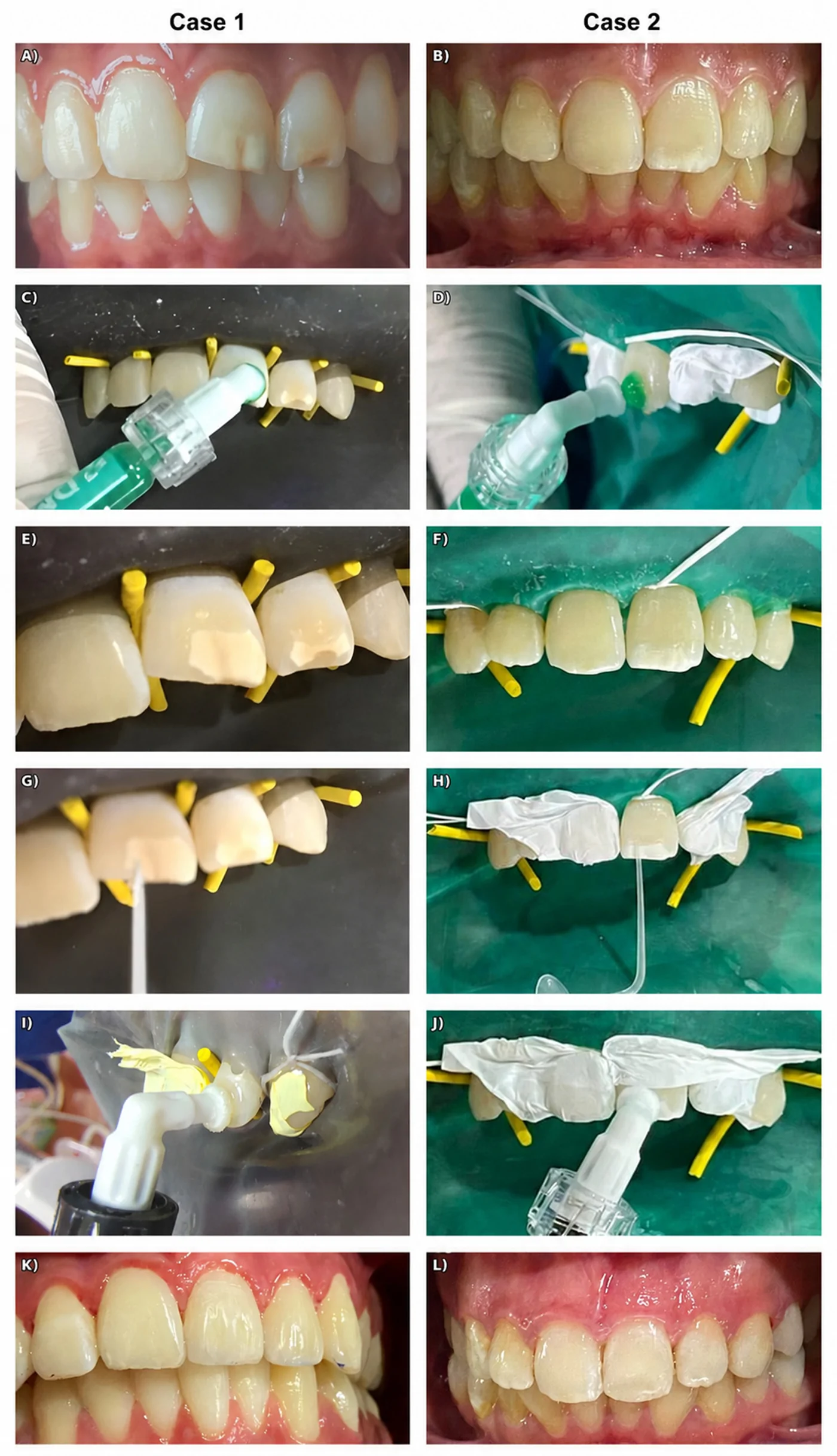
Comparative clinical sequence of cases 1 and 2 during esthetic management. **(A,B)** Pretreatment clinical appearance of Case 1 and Case 2, respectively. **(C,D)** Application of the etching agent to the affected enamel surfaces. **(E,F)** Clinical appearance following etching and rinsing. **(G,H)** Ethanol-based drying and optical reassessment before resin infiltration. **(I,J)** Application of the resin infiltrant to the conditioned enamel surfaces. **(K,L)** Final clinical appearance after completion of treatment, demonstrating improvement in enamel opacity, surface contour, and esthetic integration.

Clinical examination revealed localized whitish-brown enamel defects involving the incisal third of teeth 21 and 22 with well-defined discoloration and irregularity of the incisal contour (Figure 1A). Tooth 21 showed greater loss of incisal tooth structure consistent with an uncomplicated enamel-dentin crown fracture, whereas tooth 22 showed a limited enamel-only incisal defect. No obvious cavitation or extensive facial enamel loss was evident. The surrounding gingival tissues appeared clinically healthy. Periodontal probing depths and mobility were within physiological limits, and the teeth were not tender to percussion or palpation.

### 2.2 Case 2

An 18-year-old male patient presented with dissatisfaction regarding whitish-brown discoloration and fracture of the maxillary anterior teeth. The discoloration had been present since eruption. He reported a fall during childhood and another fall 4 months before presentation. The medical history was non-contributory, with no current medication use or known allergies. There was no relevant family history of developmental enamel defects or previous dental treatment involving the affected teeth.

Clinical examination revealed localized whitish-white to light-brown enamel changes involving teeth 21 and 22, associated with irregularity of the incisal edges (Figure 1B). Tooth 21 showed a greater incisal structural defect, whereas tooth 22 showed a more limited enamel defect. No obvious cavitation or extensive facial enamel loss was evident. The surrounding gingival tissues appeared clinically healthy, without visible swelling or inflammation. Periodontal findings were within physiological limits, and there were no clinical features suggestive of acute pulpal or periodontal pathology.

## 3 Timeline

The episode of care for both patients is summarized in Table 1.

**Table 1:** Timeline of presentation, intervention and follow-up for both cases.

Time point	Case 1	Case 2
Relevant history	Childhood history of falls; discoloration and fracture visible since eruption of permanent incisors.	Childhood fall and another fall 4 months before presentation; discoloration visible since eruption.
Clinical examination	17-year-old male; whitish-brown opacity of teeth 21 and 22; Ellis Class II fracture in 21 and Ellis Class I fracture in 22; no pain, swelling, or sensitivity.	18-year-old male; whitish-brown opacity of teeth 21 and 22; Ellis Class II fracture in 21 and Ellis Class I fracture in 22; esthetic concern.
Diagnostic assessment	Localized trauma-associated developmental enamel defect involving teeth 21 and 22 with associated uncomplicated incisal fractures.	Localized trauma-associated developmental enamel defect involving teeth 21 and 22 with associated uncomplicated incisal fractures.
Microabrasion	Two 1-minute cycles of microabrasion were performed using 6.6% hydrochloric acid with silicon carbide microparticles (Opalustre; Ultradent Products Inc., South Jordan, UT, USA), with water rinsing between applications. Mild reduction in discoloration was observed.	Two 1-minute cycles of microabrasion were performed using 6.6% hydrochloric acid with silicon carbide microparticles (Opalustre; Ultradent Products Inc., South Jordan, UT, USA), with water rinsing between applications. Mild reduction in discoloration was observed.
Resin infiltration	Etching and ethanol-based drying were repeated according to the optical response. Five etch-dry cycles were performed before satisfactory improvement was achieved. Resin infiltrant was applied for 5 min, light cured for 40 s, reapplied for 1 min, and light cured again.	Etching and ethanol-based drying were repeated according to the residual opacity before resin infiltration; the exact number of repetitions was not documented. Resin infiltrant was applied for 3 min, light cured for 40 s, reapplied for 1 min, and light cured again.
Restorative phase	A thin layer of G-aenial Anterior composite resin (GC Corp., Tokyo, Japan) was placed to correct the shallow post-etch surface depression and restore enamel contour, followed by finishing and polishing.	The incisal defects were restored using OMNICHROMA universal composite resin (Tokuyama Dental Corp., Tokyo, Japan), followed by finishing and polishing with Sof-Lex XT discs (3M ESPE, St Paul, MN, USA).
Follow-up and outcomes	1-week follow-up: satisfactory esthetics and contour; no sensitivity or periodontal complications.	2-month follow-up: stable color integration and morphology; no sensitivity or periodontal complications.

## 4 Diagnostic assessment

The working diagnosis in both patients was a localized trauma-associated developmental enamel defect involving teeth 21 and 22, accompanied by uncomplicated incisal fractures. The diagnosis was based on the localized distribution of the defects, their presence since eruption of the permanent incisors, the reported history of childhood trauma, and the clinical appearance of the affected enamel.

Differential diagnoses included caries-related white spot lesions, dental fluorosis, molar-incisor hypomineralization and other non-traumatic developmental enamel defects. A caries-related white spot lesion was considered less likely because of the localized distribution and developmental history of the lesions. Dental fluorosis was considered less likely because the defects were localized rather than generalized or symmetrically distributed. Molar-incisor hypomineralization was also considered because of the enamel opacity; however, the localized distribution and strong temporal association with childhood trauma were more consistent with a trauma-associated developmental enamel defect. The history of trauma affecting the primary anterior region and the subsequent localized defect in the permanent successors supported a trauma-associated developmental origin.

The enamel defects were further assessed clinically during treatment according to their response to surface conditioning and ethanol-based drying. Persistence of opacity after drying indicated the need for additional surface conditioning before resin infiltration. The prognosis was considered favorable for improvement in esthetic appearance, although long-term monitoring of color stability, surface integrity, and composite restoration was advised.

## 5 Therapeutic intervention

Both cases were managed using a staged sequence of enamel microabrasion, resin infiltration, and selective composite restoration.

### 5.1 Isolation and enamel microabrasion

Rubber dam isolation was used, with Teflon tape protection where required. Enamel microabrasion was performed using 6.6% hydrochloric acid slurry containing silicon carbide microparticles (Opalustre; Ultradent Products Inc., South Jordan, UT, USA). The material was applied to the affected labial enamel surfaces using a rubber polishing cup on a slow-speed contra-angle handpiece for 1 min per cycle, followed by water rinsing. Two cycles were performed in each case, producing a mild reduction in discoloration.

### 5.2 Resin infiltration

Resin infiltration was performed using a low-viscosity resin infiltrant (Icon; DMG, Hamburg, Germany) according to the manufacturer's instructions. The affected enamel surfaces were etched with Icon-Etch for 2 min, rinsed for 30 s, and dried with Icon-Dry for 30 s to assess the optical response. The etch-dry sequence was repeated when residual opacity persisted. Representative stages of etchant application, post-etch appearance, and ethanol-based optical assessment in both cases are shown in Figures 1C–H**.**

In Case 1, five etch-dry cycles were performed before satisfactory optical improvement was achieved. In Case 2, etching and ethanol-based drying were repeated according to the residual opacity before resin infiltration; the exact number of repetitions was not documented.

In Case 1, the infiltrant was applied for 5 min and light cured for 40 s, followed by a second 1-minute application and light curing. In Case 2, the infiltrant was applied for 3 min and light cured for 40 s, followed by a second 1-minute application and light curing.

### 5.3 Restorative phase

In Case 1, repeated surface conditioning resulted in a shallow enamel surface depression. A thin layer of G-ænial Anterior composite resin (GC Corp., Tokyo, Japan) was therefore placed selectively to restore the surface contour, followed by finishing and polishing.

In Case 2, composite restoration was indicated primarily to reconstruct the pre-existing incisal defects. OMNICHROMA universal composite resin (Tokuyama Dental Corp., Tokyo, Japan) was placed using a direct layering technique, followed by finishing and polishing with Sof-Lex XT discs (3M ESPE, St Paul, MN, USA).

## 6 Follow-up and outcomes

In Case 1, immediate improvement in enamel opacity and optical integration was observed following resin infiltration and selective composite restoration. At the 1-week review, the surface contour and esthetic integration remained satisfactory. The patient reported no postoperative sensitivity or pain, and no adverse periodontal findings were identified.

In Case 2, immediate improvement in enamel opacity and esthetic blending was observed following infiltration and restoration of the incisal defects. At the 2-month review, the improvement in color integration and incisal morphology remained clinically satisfactory. No postoperative sensitivity, pain, or periodontal complications were reported.

Both patients expressed satisfaction with the esthetic improvement and reported no functional discomfort during the available follow-up period. The different follow-up durations between the two cases were considered when interpreting the outcomes.

## 7 Discussion

The present cases demonstrate a staged esthetic approach for localized trauma-associated developmental enamel defects of the maxillary anterior teeth. Both patients presented with persistent whitish-brown enamel abnormalities and associated incisal structural defects following childhood trauma. Microabrasion produced only mild improvement, after which resin infiltration was used for optical masking and selective composite restoration was performed where structural correction was required. Both patients showed satisfactory short-term esthetic improvement without postoperative sensitivity or periodontal complications.

Trauma to primary anterior teeth may interfere with development of the permanent successors and result in localized enamel opacity, hypoplasia, or structural defects. In the present cases, localization to the maxillary anterior teeth, persistence since eruption, childhood trauma history, and associated incisal defects supported a trauma-associated developmental origin. Distinguishing qualitative opacity from quantitative hypoplasia remains clinically relevant because lesion morphology and structural loss influence treatment selection.

Resin infiltration improves optical masking by replacing air or fluid within enamel microporosities with a low-viscosity resin, thereby reducing the refractive-index difference and light scattering within the lesion (8–10). Adequate surface conditioning facilitates penetration of the infiltrant, and hydrochloric acid has been shown to remove the resistant surface layer more effectively than phosphoric acid (11). The optical response following etching and ethanol-based drying was therefore used to guide further conditioning before infiltration (12). Five etch-dry cycles were documented in Case 1, whereas in Case 2 the sequence was repeated according to residual opacity, although the exact number of repetitions were not recorded. The mechanism and clinical principles of resin infiltration for enamel lesions have also been described previously.

Clinical studies have reported sustained esthetic improvement following resin infiltration of postorthodontic and arrested white spot lesions (13, 14). Resin infiltration has also demonstrated better esthetic improvement than microabrasion alone for suitable postorthodontic lesions (15). Systematic review evidence supports its lesion-masking effect while emphasizing appropriate case selection and the need for longer follow-up (16). These findings support the staged approach used in the present cases, where microabrasion was initially employed for the superficial component and infiltration was subsequently used when discoloration persisted.

Direct composite restoration addressed structural deficiencies that could not be corrected by optical masking alone. In Case 1, a thin composite layer was required to restore contour after a shallow surface depression became evident following repeated conditioning. In Case 2, composite restoration primarily reconstructed the pre-existing incisal defect. Thus, infiltration and composite restoration served complementary purposes, addressing optical alteration and structural loss, respectively.

Long-term color stability is an important consideration because resin-infiltrated enamel may be susceptible to extrinsic staining. Experimental evidence has demonstrated staining potential following exposure to chromogenic substances (17, 18) whereas long-term clinical follow-up has shown acceptable color stability when finishing and maintenance are adequate (19). Finishing, polishing, and periodic review were therefore emphasized in both cases.

Similar case reports have described the combined use of microabrasion, resin infiltration, bleaching, and direct composite restoration for enamel hypoplasia and anterior esthetic rehabilitation (20, 21). The present cases further illustrate that treatment may be individualized according to lesion response rather than relying on a single technique.

The main strength of this report is the staged treatment strategy, in which microabrasion, optical reassessment, infiltration, and composite augmentation were applied according to clinical need. Limitations include the small number of patients, short and unequal follow-up periods, absence of objective colorimetric assessment, and lack of quantitative evaluation of lesion depth or enamel thickness. The exact number of etch-dry repetitions in Case 2 was also not documented, and repeated conditioning in Case 1 resulted in a shallow enamel depression requiring selective composite correction.

Overall, localized trauma-associated developmental enamel defects may be managed using a staged approach in which microabrasion addresses superficial discoloration, resin infiltration improves subsurface optical masking, and limited composite restoration corrects structural deficiencies. Longer follow-up is required to assess color stability, restoration integrity, and patient satisfaction.

## 8 Patient perspective

Both patients reported that the discoloration and irregular appearance of their maxillary anterior teeth had been an esthetic concern before treatment. Following treatment, both reported improvement in the appearance of the affected teeth and satisfaction with the outcome. Neither patient reported postoperative sensitivity or functional discomfort during the available follow-up period. They were advised regarding oral hygiene, periodic clinical review, polishing when indicated, and the possibility of future maintenance of the composite restorations.

## Data Availability

All relevant data supporting the findings of this report are included within the article. Additional data are available from the corresponding author upon reasonable request.
